# Recumbent Position during Syncope

**Published:** 1854-11

**Authors:** 


### THE EFFECTS OF THE RECUMBENT POSITION DURING SYNCOPE, PHYSIOLOGICALLY CONSIDERED.

Mr. Richardson read before the Physiological Section of the Medical Society of London (April 20, 1854,) a paper on this subject. The author commenced by stating that though the fact, that the recumbent or horizontal posture often affords marked and immediate relief in syncope, is generally admitted, no very distinct attempt had hitherto been made to explain the principles on which it acted. One view, however, had fixed itself in the professional mind, and required to be carefully refuted. This view is, that the horizontal posture relieves syncope, by allowing the blood to gravitate to the brain and medulla, so that these centres, gaining energy by this process, react on the heart and supply it with new vigor. This theory had been supported by many writers, among whom the author quoted Dr. Alison, of Edinburgh, Dr. Ash, and Sir George Lefevre. The latter author relates a case in which syncope occurred on the patient assuming the erect position. It was found to be connected with the presence of varicose veins in the leg, and was prevented by the application of bandages. In this case, Mr. Richardson observed, that the brain being deprived of blood was secondary to the fact that the propelling power of the heart was to a great extent lost through the mechanical impediment in the course of the circulation—an impediment which the bandages relieved. It was also obvious that the blood detained in the lower parts of the body could not reach the brain without first passing through the heart. Moreover, any renewed force which the heart might receive from the nervous centres would be quite useless until it contained blood on which to act. When we perform transfusion, we do so for the purpose of filling the heart with its natural stimulus, not for the immediate purpose of exciting the nervous centres. The recovery of consciousness on laying a person in the supine position is no proof of the correctness of the hypothesis above mentioned; for, when consciousness ceases during syncope, it ceases as a consequence of failure of the circulation, and returns in proportion as the circulation becomes re-established. Mr. Richardson had observed that the first symptom of recovery from syncope invariably was the return of the heart's beat, and that then muscular motion, consciousness, and animal heat followed. Again, in some instances, the function of the heart fails, while the functions of the nervous system remain perfect; and, on the other hand, the manifestations of the nervous system may be suspended by narcotic poisons, while the heart continues to beat with power. There may also be extensive disease of the cerebro-spinal axis, and yet the heart's action remains unaffected. Again, in the animal kingdom, the size of the heart and activity of the circulation bear no relation to the development of the nervous system; and, in the formation of the vertebrate embryo, the heart begins to pulsate before it is connected with any nervous centres. Mr. Richardson next proceeded to offer his own theory of the manner in which the recumbent position produces recovery from syncope. The explanation appealed to mechanical laws, and was very simple. It must be remembered, that the arterial blood sent from the heart first ascends, and that the venous blood descends from the upper and ascends from the lower parts. When blood is withdrawn from the upper part of the erect body, the heart loses its power of sending the blood along the aorta; hence the blood, losing the *vis a tergo*, gravitates in the veins in the lower half of the body. At the same time, the heart not having sufficient power to propel the blood to the brain and other parts, consciousness is lost, and voluntary motion and the production of animal heat fail. Death would now soon occur, from the heart ceasing to pulsate, and from the blood coagulating in the veins; but the body falls, or is laid down, and then the blood contained in the veins of the lower part of the body is poured into the heart, and again it excites to contraction. Thus the whole circulation is restored, and the brain and every part of the body receiving a fresh supply of blood, resume their proper functions; but to no one of these parts is due the least credit for having restored the movements of the heart. When blood is withdrawn from the lower part of the body, the chances of recovery are much lessened; for what was in the former case a reservoir, now becomes a running cistern. The recumbent position is here equally valuable, since it leads to a distribution of blood through the vessels above the heart. It might be even an advantage to put the head, in these cases, slightly lower than the trunk, until the cause of the hemorrhage was removed.—But, in general, the recumbent position is all that is required. The manner in which the killing of calves is performed in slaughter-houses, was adduced by Mr. Richardson as an instance of the effects produced by position on the loss of blood. He next proceeded to speak of syncope dependent on an over-burdened condition of the heart, or on debility of the cardiac walls. In these cases, the recumbent position enables the blood to pass more readily into the pulmonary artery and aorta, while the venous circulation generally is rendered more equable. Mr. Richardson then referred to several experiments which proved to demonstration the truth of his theory. Having slowly narcotized a kitten, he laid bare the heart by a careful dissection, without opening the right pleural cavity; he then punctured the arteria innominata, while the animal was suspended by the head. The heart continued contracting for some minutes, but at last the right auricle collapsed, and pulsation ceased. At this moment the body of the animal was reversed, and suspended by the heels. The auricle instantly refilled from the inferior cava, and the heart resumed its contractions. This was repeated with the same results. On another occasion, the vena cava inferior was tied previous to the reversion of the body, when no reaction took place until the ligature was removed. In a third experiment, the animal was suspended, in the first place, by the heels, and, the abdominal aorta being punctured in the middle, the auricle was allowed to collapse as before; the animal was then turned head upwards, when the auricle filled from the superior venous trunks.—There could be no doubt as to the results of these experiments.—*Med. Times and Gaz.*, April 22, 1854.

